# Policy vs. Practice: Nutritional Quality and Menu Structure in Polish Hospitals During the “Good Meal in Hospital” Pilot Program—A Multicenter Cross-Sectional Study

**DOI:** 10.3390/nu18071034

**Published:** 2026-03-25

**Authors:** Agnieszka Orkusz, Martyna Orkusz

**Affiliations:** 1 Department of Biotechnology and Food Analysis, Wroclaw University of Economics and Business, 53-345 Wroclaw, Poland; 2 Faculty of Biotechnology and Food Science, Wroclaw University of Environmental and Life Sciences, 50-375 Wroclaw, Poland; 125360@student.upwr.edu.pl

**Keywords:** hospital nutrition, hospital meals, standard diet, menu analysis, patient care, dietary quality, pilot program evaluation

## Abstract

**Background**: Hospital nutrition is an essential component of patient care; however, the nutritional quality of meals in Polish hospitals has raised concerns for many years. In response to these challenges, the Ministry of Health implemented a nationwide pilot program, “Good Meal in the Hospital,” to improve nutritional quality by developing and implementing a model tailored to patients’ needs. This study aimed to assess the compliance of hospital-standard diets with the program’s assumptions. **Methods**: Ten-day menus from ten hospitals across different regions of Poland (a total of 100 menus) were analyzed. A quantitative assessment of energy and nutritional values was conducted by calculating the average daily intake of energy and selected nutrients (protein, carbohydrates including sugars (mono- and disaccharides), fat, saturated fatty acids, fiber, and salt) and comparing these with national recommendations for hospital diets for adults. In parallel, a qualitative assessment of ten menu structure criteria was performed using a binary scoring system (0/1). **Results**: None of the analyzed hospitals met all quantitative and qualitative criteria simultaneously. All hospitals complied with recommendations for protein and carbohydrate content, whereas the most frequent deviations concerned excessive intake of fat, saturated fatty acids, and salt. Qualitative scores ranged from 6 to 10 points, with the most significant shortcomings related to the frequency of legumes and fish. A particularly noteworthy finding was the discrepancy between qualitative and quantitative compliance of the menus. **Conclusions**: The fact that none of the analyzed hospitals met all program criteria simultaneously indicates the limited effectiveness of its implementation in its current form.

## 1. Introduction

The effectiveness of patient treatment in hospitals is determined not only by the proper therapeutic process conducted by medical personnel but also by adequate patient nutrition and nutritional education [1,2]. Ensuring appropriate hospital nutrition, a key component of patient care, has long remained a significant challenge in Poland. Meals served in hospitals were often of low quality and frequently failed to meet established norms and recommendations [3,4,5].

A report by the Supreme Audit Office (SAO) [6]—Poland’s highest governmental audit body responsible for evaluating public institutions, including healthcare facilities—revealed that in most audited hospitals, patients received meals inappropriate for their health status, prepared from low-quality raw materials, and characterized by inadequate nutritional value. Analyses of hospital meal samples conducted on behalf of SAO by the State Sanitary Inspection and Trade Inspection revealed deficiencies in nutrients that could lead to serious health consequences, such as demineralization, softening and deformation of bones, anemia, tissue hypoxia, excessive muscle excitability, tremors and painful muscle cramps, anxiety, insomnia, hypertension, and impaired kidney function.

Most hospitals also showed excessive salt content in meals (ranging from 142% to 374% of the standard), as well as either underestimated (3–28%) or overestimated (13–50%) caloric content. Patients were served too few vegetables, fruits, and seafood, and too many fatty meat products and lower-quality cold cuts. Meals were rich in saturated fatty acids, included canned or farmed fish from polluted areas, and often contained substitutes for menu items—for example, “milk fat spread” instead of “butter,” or dried protein hydrolysate with cheese flavor and aroma instead of “cheese.” As a result, hospital food did not fulfill its basic function—supporting the treatment and recovery process—and in some cases could even be harmful.

The SAO report also highlighted systemic problems, including unclear daily nutrition rates (9.55–17.99 PLN per patient per day), the lack of legal standards for hospital meals, the absence of procedures for monitoring catering services, and the lack of an obligation to employ dietitians.

In response to these irregularities, the Ombudsman for Civil Rights [7] appealed to the Minister of Health for systemic regulation of patient nutrition. It was emphasized that hospitals often limit nutrition budgets for economic reasons, and that 70% of catering companies preparing meals for hospitals used expired products, failed to inform about allergens, used cheaper substitutes, and provided improperly portioned meals.

The results of the 2023 inspection by the Wroclaw Voivodeship Sanitary-Epidemiological Station [8], which covered 72 hospitals, confirmed the persistence of numerous irregularities, especially low content of vegetables, fruits, and fish; deficiencies in milk and dairy products; absence of legumes and whole-grain cereal products (whole groats, whole-grain flakes); excessive use of highly processed foods; and unclear labeling of dishes, allergens, and cooking methods.

Malnutrition in healthcare settings leads to severe clinical and economic consequences, such as prolonged hospitalization and increased treatment costs [9,10,11,12]. Therefore, as public trust institutions, hospitals should promote healthy eating habits rather than perpetuate harmful patterns resulting from poorly conducted nutrition.

In response to long-standing systemic problems, on 25 September 2023, the Ministry of Health launched the nationwide pilot program “Good Meal in the Hospital” [13]. The program aimed to increase access to nutritional advice, implement a patient-centered nutrition model, and establish measurable indicators to evaluate its effectiveness and enable its expansion across the entire Polish healthcare system [13]. The pilot program was initially scheduled to run until 31 December 2024 [13], later extended to 30 September 2025 [14], and subsequently to 31 December 2025 [15].

An integral part of the program was organizational requirements, including the employment of a dietitian for at least half-time (4 h per day) and ensuring complete transparency of the nutrition process. Hospitals were required to publish 7-day menus, including information on meal type and energy and nutritional values (protein, total carbohydrates, including sugars (mono- and disaccharides), total fat, including saturated fatty acids, fiber, salt), preparation methods, and allergen content. Additionally, hospitals had to post daily photos of at least two meals to enable ongoing monitoring of food quality by patients, their families, and the public. The pilot was financed by providing hospitals with an additional 25.62 PLN per patient per day, covered by the program, to supplement existing nutrition funding.

It should be emphasized that scientific literature on the extent to which hospitals implement the “Good Meal in the Hospital” program is limited. Existing studies [11] indicate considerable variability across institutions and persistent irregularities, particularly in saturated fatty acids, fiber, salt content, and the provision of fish and legume seeds. Further analyses are therefore needed to assess whether the program genuinely improves patient nutrition.

Accordingly, this study aimed to evaluate the compliance of standard hospital diets with the assumptions of the “Good Meal in the Hospital” program, developed on behalf of the Ministry of Health [13]. Ten-day menus from ten hospitals across different regions of Poland were analyzed. It was hypothesized that the menus offered to patients do not comply with the program’s recommendations.

From 1 January 2026, the pilot “Good Meal in the Hospital” program was replaced nationwide by mandatory standards for hospitalized patient nutrition, as defined in the Regulation of the Minister of Health on Requirements for Hospital Nutrition [16]. The introduction of these standards made hospital nutrition part of guaranteed healthcare services, and its financing was incorporated into the overall pricing of medical procedures—in contrast to the pilot, which had a separate, targeted subsidy. This change has sparked controversy among healthcare administrators, as including nutrition costs in a general tariff may force financially weaker facilities to compete for resources between nutrition and life-saving treatments and procedures. Under such conditions, the priority is often given to emergency interventions over preventive nutrition, which may result in merely formal, rather than substantive, compliance with applicable requirements.

## 2. Materials and Methods

The study material consisted of ten 10-day menus (covering 10 consecutive days) for the standard diet, obtained from the websites of 10 hospitals in Poland participating in the “Good Meal in the Hospital” program. In total, 100 menus were analyzed (10 hospitals × 10 days). Hospitals were coded as H1 to H10, and their locations were presented at the voivodeship level: H1—Kuyavian-Pomeranian, H2—West Pomeranian, H3—Lesser Poland, H4—Masovian, H5—Pomeranian, H6 and H9—Lower Silesian, H7—Podlaskie, H8—Subcarpathian, H10—Opole. The study period covered November 2025. The choice of the autumn period was justified by the possibility of assessing the availability of fresh vegetables and fruits outside the peak growing season.

The study sample was purposively selected from hospitals participating in the “Good Meal in the Hospital” pilot program, based on predefined inclusion and exclusion criteria. Only hospitals with sufficiently complete, comparable, and publicly available menu data were eligible for inclusion, enabling both quantitative and qualitative assessments. Thus, the analysed sample was not randomly selected. The pilot program covered 582 healthcare entities [17]; therefore, the 10 hospitals included in this study should be regarded as a small purposive sample rather than a nationally representative one.

Inclusion criteria comprised the publication of menus covering ten consecutive days on the hospital website, the availability of data on daily totals of energy and nutrients (protein, carbohydrates, including sugars (mono- and disaccharides), fat including saturated fatty acids, fiber, and salt), more than three meals per day, information on the type of meals and their composition, description of cooking techniques, availability of photographs of meals for the standard diet on the hospital website, and indication of the person responsible for planning nutrition or diet. Exclusion criteria included menus that were inaccurate or illegible, lacked specified ingredient weights, contained only general descriptions (e.g., “cheese sandwich”), or did not provide information on the required nutrient content.

Differences in food service organisation were observed across the analysed hospitals. In-house kitchens operated in H1, H2, H3, H4, and H7, catering was used in H6, H8, and H10, whereas for H5 and H9, the organisational model could not be determined unequivocally.

For each hospital, based on the 10-day menus, the average daily energy value and the content of protein, carbohydrates (including sugars: mono- and disaccharides), fat (including saturated fatty acids), fiber, and salt were calculated. The obtained values were compared with the requirements for a standard adult diet presented in the document package “Nutrition Standard—Nutrition for Health” published by the Ministry of Health [18], developed by the National Institute of Public Health—National Research Institute (PZH) in cooperation with the Institute of Mother and Child and the Polish Society for Parenteral, Enteral Nutrition and Metabolism. This package includes, among other things, the characteristics of diets, recommended and contraindicated foods, and reference energy and nutrient values for hospital diets for adults.

In parallel, a qualitative assessment of the menus was conducted using a binary point scale (0/1), verifying the presence or absence of elements required by the Minister of Health’s regulations and program recommendations [18]. Ten criteria were evaluated, including the number of meals (3–5 per day), flavor and technological variety, addition of a beverage to each meal (sugar content ≤ 10 g/250 mL), presence of vegetables and fruits in each meal (minimum 400 g/day), whole-grain products (minimum once per day), presence of dairy or fortified plant-based substitutes (minimum twice per day), a protein source for each day (e.g., meat, eggs, legumes), presence of legume seeds (minimum three times in 10 days), fish, preferably marine (minimum three times in 10 days), and cooking techniques, with frying limited to a maximum of three times in 10 days. Additionally, the availability of photographic documentation of meals was analyzed as a transparency measure introduced within the pilot program.

### Statistical Analysis

The results were presented as the mean ± standard deviation. Analysis of variance (ANOVA) was employed to assess variations in the energy value and nutrient content of the hospital menus. When a significant effect was detected, the mean values were further examined using Tukey’s multiple-range test to identify specific differences. The experimental data were analyzed using Statistica version 13.3 (TIBCO Software Inc., Palo Alto, CA, USA) [19], with differences considered significant at a probability level < 0.05.

Ethical Statement: Ethical review and approval were waived because this study was based solely on the evaluation of hospital menus. No human participants were involved, and no personal or health-related data were collected.

## 3. Results

### 3.1. Quantitative Analysis of Menus (Assessment of Energy and Nutritional Value)

**Figure 1:**
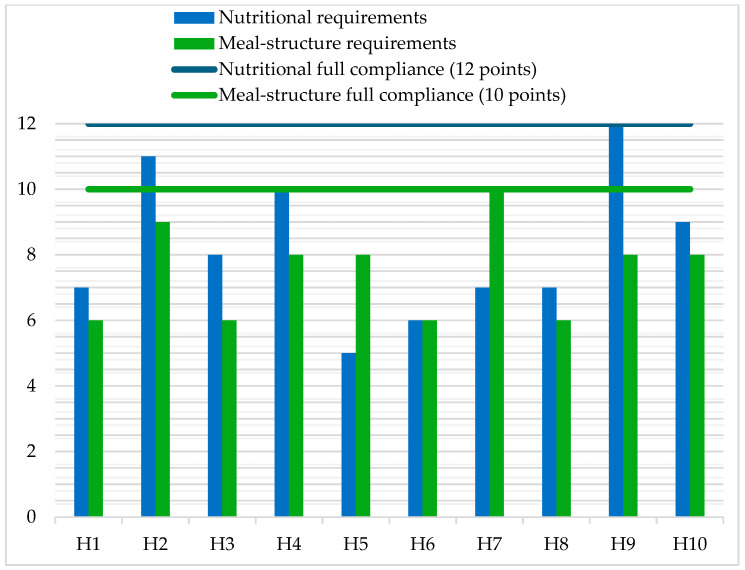
Comparison of nutritional and menu structure compliance scores across hospitals.

Despite the implementation of uniform guidelines and additional funding under the nationwide pilot program “Good Meal in the Hospital,” analysis of the menus showed that none of the ten hospitals analyzed simultaneously met all program criteria (Table 1, Table 2 and Table 3; Figure 1). Significant differences were observed between facilities in the energy and nutrient content of the menus (Table 1 and Table 2). At the same time, variation was observed in the extent to which qualitative program requirements were met across hospitals (Table 3). The results confirmed that the analyzed menus were not fully compliant with the program recommendations. The hypothesis that the menus would not conform to the program guidelines was fully confirmed.

In all analyzed facilities, the menus met the recommendations only for protein and carbohydrate intake, both in grams per day and as a percentage of total energy (Table 1 and Table 2). Noncompliance, however, concerned energy value, other nutrients, and salt. The energy value exceeded the upper limit of the standard (2400 kcal) in hospitals H3 (2562.55 kcal), H5 (2652.12 kcal), and H6 (2575.80 kcal) (Table 1). Excessive fat intake was also observed, both in g/day (H1, H3, H5, H6) and as a percentage of energy (H1, H3, H5, H6, H7, H8), as well as excessive intake of saturated fatty acids (SFA)—in g/day (H1, H4, H5, H6, H7, H10) and as a percentage of energy (H1, H4, H5, H7, H10). Particularly high SFA content was recorded in hospital H5 (38.13 g/day) (Table 1). In the same facility (H5), the salt content of meals was 12.83 g/day, representing 256.6% of the recommended value (i.e., exceeding the recommendation by 156.6%). Exceedance of the recommended 5 g/day was also observed in five other hospitals (H1, H3, H6, H7, H8). Excessive contents of sugars (mono- and disaccharides) were noted in three facilities (H2, H5, H8). Fiber content, compared to the recommendation (15 g/1000 kcal), was above the recommended level in one hospital (H10) and below the recommended level in three hospitals (H6, H7, H8) (Table 1 and Table 2).

### 3.2. Qualitative Analysis of Menus

The qualitative assessment of the 10-day hospital menus revealed considerable variability in compliance with the program’s criteria, with total scores ranging from 6 to 10 points (Table 3). The highest level of adherence to all ten quality requirements was observed in facility H7 (10/10 points), while the lowest scores (6/10 points) were recorded for hospitals H1, H3, H6, and H8.

In all analyzed facilities, the menus met four program requirements: ensuring 3–5 meals per day, maintaining adequate diversity in taste, color, and texture, including at least one daily serving of a protein source (meat, eggs, legumes, or plant-based alternatives), and applying cooking techniques that limited frying to no more than three times during the 10-day cycle.

For the remaining six quality parameters, deviations were observed. Beverages accompanying each meal were provided in 9 of 10 facilities, with noncompliance noted at hospital H8. The requirement to include vegetables and fruits with each meal, ensuring a minimum of 400 g/day, was met by six facilities, while four hospitals (H1, H6, H8, and H10) did not meet this criterion. Shortages of whole-grain products were identified in four hospitals (H3, H5, H7, H9). The requirement to provide dairy or fortified plant-based alternatives at least twice daily was met by seven hospitals, with noncompliance in H1, H3, and H8. Inadequate provision of legumes and fish, which should be served at least three times over the 10 days, was observed in seven hospitals (H1, H2, H3, H4, H5, H6, and H8) and six hospitals (H1, H3, H4, H5, H6, and H10), respectively (Table 3).

The comparison of quantitative and qualitative assessments of the menus (Figure 1) showed that high compliance with qualitative requirements did not always correspond to proper nutritional balance. An example of such a discrepancy was hospital H5, which, despite achieving a relatively high qualitative score (8/10 points), demonstrated the lowest nutritional compliance (5/12 parameters), due in part to exceeding recommendations for sodium and saturated fatty acids (Table 1). Conversely, facility H7 achieved full compliance with qualitative requirements (10/10 points) but only moderate compliance with the quantitative assessment (7/12 parameters). Hospitals H2 and H9 showed high scores in both dimensions, reflecting the most comprehensive implementation of the pilot program’s objectives.

## 4. Discussion

The results of this study indicate significant discrepancies between the objectives of the nationwide pilot program “Good Meal in the Hospital” and their implementation in practice. Despite additional funding, standardized guidelines, and menu transparency, none of the ten analyzed hospitals achieved full compliance with all quantitative and qualitative program criteria, confirming the research hypothesis that implementing nutrition standards is challenging.

The assessment showed that in all hospital facilities, protein and carbohydrate intake were fully compliant with the standards (Table 1). Adequate protein intake is crucial for immunity and reducing susceptibility to infections, which is important during hospitalization [20]. Surgical procedures and severe systemic illness often induce insulin resistance. Excess carbohydrates or improper timing (e.g., serving overly large portions at once) may exacerbate hyperglycemia and negatively affect treatment outcomes [21].

At the same time, deviations were observed in the analyzed menus regarding energy content and other nutrients (Table 1 and Table 2). In three facilities (H3, H5, H6), energy intake exceeded the upper limit of the recommended range (2400 kcal/day) (Table 1), which, under hospitalization conditions, often with limited activity and coexisting metabolic disorders, may lead to excessive energy intake relative to some patients’ needs. Adequate energy intake protects against the development of overweight and obesity, which are direct risk factors for numerous metabolic and cardiovascular disorders [22]. Excess body weight significantly increases the likelihood of hypertension, type 2 diabetes, and cerebrovascular diseases [23]. Moreover, epidemiological studies confirm a strong correlation between obesity and increased incidence of certain cancers, including breast, pancreatic, prostate, and bladder cancers [24,25].

The most frequently observed irregularities in the analyzed hospital menus were excessive intake of saturated fatty acids (in seven facilities) and excessive intake of total fat and salt (in six facilities) (Table 2). This finding is significant because a diet high in fat, especially saturated fatty acids, is associated with increased risk of coronary heart disease and the development of certain cancers, particularly colorectal and breast cancer [26,27,28].

Additionally, excessive salt intake is a well-documented risk factor for hypertension and cardiovascular complications, which is particularly relevant in the hospitalized population, often burdened with chronic comorbidities [29]. The problem of excessive salt intake in the Polish population is systemic, with improper consumption patterns observed from an early age [30]. This indicates a widespread issue in Polish institutional catering, characterized by the overuse of processed products (e.g., cured meats, cereal products), directly contributing to exceeding recommended salt intake.

Excessive contents of mono- and disaccharides were noted in the menus of three of the ten analyzed hospitals (H2, H5, H8) (Table 1 and Table 2), likely due to the high proportion of foods rich in these sugars, such as jams and sweetened beverages. Fiber intake was both below recommendations (H6, H7, H8) and above recommendations (H10). A particularly unfavorable pattern was observed in H8, where high sugar content coincided with low fiber. Adequate fiber intake, by regulating glucose absorption rates and influencing lipid metabolism, shapes the metabolic quality of the diet. Maintaining a fiber deficit alongside excessive intake of sugars (mono- and disaccharides) during hospitalization contradicts current guidelines for cardiometabolic disease prevention and optimization of treatment [31,32].

Substantial variation in the overall qualitative assessment (6–10 points) indicates that the degree of program implementation was highly facility-dependent, with full compliance with all qualitative criteria achieved in only one hospital (H7) (Table 3). Irregularities related to the infrequent serving of fish and legume seeds (Table 3) are particularly noteworthy, as they concern food groups of key importance in a health-promoting dietary model.

The most significant challenges in program implementation involved the regular provision of legume seeds and fish, as well as—in some facilities—adequate provision of vegetables and fruits, whole-grain products, and dairy/fortified alternatives (Table 3). This pattern of noncompliance indicates that formal implementation of the standard did not always translate into full achievement of its nutritional goal: improving patient diet quality by increasing the proportion of high-nutrient foods. At the same time, menu analysis showed that fish and legume seeds were included in diverse forms. Fish dishes included breaded fillets, fish meatballs, fish with vegetables in aspic, fish pastes (tuna and salmon), fish salads (including spicy mackerel salad), Greek-style fish, and herring in tomato sauce. Legume sources were mainly served as soups (e.g., with beans, peas, or chickpeas), spreads and pâtés (from lentils, chickpeas, soy), and dishes such as “fasolka po bretońsku.” Jodczyk et al. [11] also reported difficulties in ensuring adequate provision of fish and legume seeds during the evaluation of the implementation of the “Good Meal in the Hospital” program for diabetic patients.

The statistically significant differences observed between hospitals in the energy and nutrient content of menus indicate that the quantitative implementation of the pilot program varied significantly across institutions (Table 1). The results also indicate that there were differences between hospitals not only in the extent but also in the type of observed irregularities. For example, hospitals H3, H5, and H6 simultaneously exceeded the recommended daily energy value and had excessive fat content. Hospital H5 was further distinguished by having the highest saturated fatty acid content and salt content. Meanwhile, hospitals H2, H5, and H8 showed excessive amounts of mono- and disaccharides, while hospitals H6, H7, and H8 had insufficient fiber content. This indicates that the observed irregularities had a different nature in the respective institutions.

A significant finding in terms of program evaluation is the discrepancy between qualitative compliance (Table 3) and quantitative—energy and nutrient—compliance (Table 1 and Table 2; Figure 1). Examples of H5 (relatively high qualitative score with the lowest quantitative compliance) and H7 (full qualitative compliance with only moderate quantitative compliance) indicate that compliance with the program’s qualitative criteria does not necessarily mean that the menu is properly balanced in terms of energy and nutrient content. This discrepancy indicates that evaluation of the pilot program should simultaneously consider two dimensions: (1) compliance with the program’s qualitative criteria and (2) the actual nutritional quality of the menus. Only the combined assessment of these two dimensions allows the determination of whether program implementation is substantive rather than merely formal. Whether a diet is well-balanced depends not only on the presence of certain food groups in the menu, but also on how often they appear, what specific items within those groups are chosen, how large the portions are, and how the dishes are prepared. As a result, a menu may formally meet the program’s structural requirements—such as including certain food groups—but still fail to provide the appropriate energy and nutrient intake.

The results of this study indicate that the “Good Meal in the Hospital” program represents an important systemic step. However, its effectiveness depends on the actual quality of program implementation in day-to-day menu planning. This is particularly significant because, even during short hospital stays, healthcare facilities can shape patients’ health-promoting behaviors by providing meals of appropriate nutritional value, consistent with national guidelines for health promotion and disease prevention [33]. Moreover, studies conducted among patients with cardiovascular and oncological diseases indicate that hospitalization may foster readiness for immediate dietary changes [34,35].

### Study Limitations

It is important to note that this study has certain limitations. Menus from only ten hospitals were evaluated; therefore, the results should not be interpreted as representative of all Polish hospitals or of all healthcare entities participating in the “Good Meal in the Hospital” pilot program. The study sample was purposively selected according to predefined inclusion and exclusion criteria. The analysis concerned exclusively the standard diet and a single study period (November 2025), which may not reflect seasonal or long-term variability in menu planning. The assessment was based on published menus and declared energy and nutrient values, rather than on laboratory-verified meal composition. The analysis also did not account for potential differences between planned menus, meals actually served, and patient consumption. An additional issue requiring further investigation is whether menu quality and compliance with nutritional standards differ depending on the food service organisation model, that is, whether menu planning and meal preparation are carried out by the hospital itself or by an external catering provider. Although such organisational differences were observed among the analysed hospitals, their impact was beyond the scope of the present study and was not included as an inclusion criterion. No patient satisfaction or clinical outcome data were available; therefore, it was not possible to assess patients’ perceptions of meal quality or the potential clinical relevance of the observed nutritional shortcomings.

## 5. Conclusions

Despite the implementation of the “Good Meal in the Hospital” pilot program, none of the analyzed hospitals met all the qualitative and quantitative criteria simultaneously, indicating difficulties in meeting them in practice. These findings have important practical implications for both policymakers and hospital administrators. As demonstrated, meeting the structural requirements for menus—such as the specified frequency of serving selected food groups—does not in itself ensure an adequate supply of energy and nutrients. Therefore, it is necessary to implement an integrated monitoring system that assesses both the structural (qualitative) compliance of menus and their energy and nutritional value. For policymakers, this means that hospital financing mechanisms should be linked directly to verified, measurable parameters of nutritional quality, rather than merely to formal compliance with structural requirements. At the same time, hospital administrators should implement regular internal audits to ensure that planned menus are consistent with the principles of proper patient nutrition.

To support this transition and increase the effectiveness of hospital nutrition programs, it is strongly recommended that comprehensive training systems be introduced for catering and dietetic staff. This would enable the concurrent fulfillment of quantitative and qualitative norms, driving an overall improvement in hospital nutrition standards.

Future studies should include larger, more representative samples of hospitals, different types of diets, multiple time periods, and verification of the portions actually served and consumed by patients, to accurately assess the effectiveness of hospital nutrition standards and ensure tangible improvements in the quality of meals in Polish hospitals. Future studies should also examine whether the food service model, including in-house hospital kitchens versus outsourced catering, is associated with differences in menu quality, energy and nutrient content, and compliance with hospital nutrition standards.

## Figures and Tables

**Table 1:** Energy and nutritional value of 10-day standard diet menus in 10 hospitals.

Parameter	Recommended Standards [18]	H1	H2	H3	H4	H5	H6	H7	H8	H9	H10
Energy (kcal/day)	2000–2400	2333.76 ^b^ ± 119.94	2080.93 ^a^ ± 93.62	2562.55 ^c^ ± 184.71	2303.30 ^b^ ± 135.38	2652.12 ^c^ ± 213.22	2575.80 ^c^ ± 24.90	2216.70 ^b^ ± 46.75	2243.70 ^b^ ± 39.25	2254.77 ^b^ ± 140.18	2100.80 ^a^ ± 70.37
Protein (g/day)	25–50 g/1000 kcal (50–120 g/2000–2400 kcal)	101.40 ^b^ ± 12.03	82.04 ^a^ ± 12.66	109.93 ^c^ ± 8.65	111.90 ^c^ ± 11.58	113.91 ^c^ ± 9.39	98.70 ^b^ ± 2.00	97.12 ^b^ ± 4.64	85.61 ^a^ ± 4.25	87.72 ^a^ ± 11.80	77.30 ^a^ ± 9.41
Protein (% of energy)	10–20%	17.13 ^b^ ± 2.18	15.75 ^a^ ± 2.13	17.21 ^b^ ± 1.53	19.48 ^c^ ± 2.18	17.26 ^b^ ± 1.90	15.32 ^a^ ± 0.34	17.53 ^b^ ± 0.87	15.27 ^a^ ± 0.84	15.53 ^a^ ± 1.57	14.72 ^a^ ± 1.74
Fat (g/day)	28–33 g/1000 kcal (56–79 g/2000–2400 kcal)	87.63 ^c^ ± 9.05±	59.34 ^a^ ± 5.07	86.81 ^c^ ± 14.85	72.30 ^b^ ± 13.70	98.15 ^d^ ± 14.21	89.40 ^cd^ ± 2.91	75.25 ^b^ ± 4.25	75.34 ^b^ ± 7.71	66.69 ^ab^ ± 13.95	66.80 ^ab^ ± 7.38
Fat (% of energy)	25–30%	33.79 ^d^ ± 2.62	25.67 ^a^ ± 1.99	30.47 ^bcd^ ± 4.43	28.08 ^a^ ± 3.90	33.23 ^bcd^ ± 3.28	31.23 ^bc^ ± 0.92	30.55 ^bc^ 1.61	30.19 ^bc^ ± 2.61	26.55 ^a^ ± 4.67	28.68 ^ab^ ± 3.66
Saturated fatty acids (g/day)	<11 g/1000 kcal (22–26.4 g/2000–2400 kcal)	28.86 ^c^ ± 3.74	18.36 ^b^ ± 3.52	20.90 ^b^ ± 4.60	28.20 ^c^ ± 5.13	38.13 ^d^ ± 6.62	28.30 ^c^ ± 2.16	28.61 ^c^ ± 2.98	22.85 ^b^ ± 1.97	12.92 ^a^ ± 4.88	28.10 ^c^ ± 3.78
SFA (% of energy)	<10%	11.13 ^e^ 1.31±	7.94 ^bc^ ± 1.50	7.35 ^b^ ± 1.81	11.01 ^e^ ± 1.85	12.97 ^e^ ± 2.16	9.89 ^d^ ± 0.74	11.62 ^e^ ± 1.22	9.16 ^cd^ ± 0.74	5.14 ^a^ ± 1.85	12.07 ^e^ ± 1.85
Carbohydrates (g/day)	113–163 g/1000 kcal (226–391 g/2000–2400 kcal)	289.32 ^a^ ± 21.84	313.33 ^ab^ ± 14.70	341.00 ^c^ ± 39.25	310.80 ^a^ ± 23.55	328.69 ^bc^ ± 34.09	344.10 ^c^ ± 6.26	300.29 ^a^ ± 10.77	346.74 ^c^ ± 26.84	319.82 ^b^ ± 35.96	306.20 ^ab^ ± 26.24
Carbohydrates (% of energy)	45–65%	49.59 ^a^ ± 3.99	60.25 ^c^ ± 1.84	53.23 ^ab^ ± 4.80	54.09 ^b^ ± 4.47	49.77 ^a^ ± 7.38	53.44 ^ab^ ± 0.84	54.19 ^b^ ± 1.51	61.84 ^c^ ± 4.98	56.81 ^b^ ± 6.07	58.25 ^c^ ± 3.77
Sugars (mono- and disaccharides) (g/day)	<25 g/1000 kcal (<50–60 g/2000–2400 kcal)	52.64 ^bc^ ± 11.47	81.45 ^d^ ± 9.84	57.69 ^c^ ± 15.46	44.05 ^ab^ ± 9.90	87.53 ^d^ ± 17.02	55.50 ^bc^ ± 7.65	48.63 ^b^ ± 7.66	107.87 ^e^ ± 13.39	59.09 ^c^ ± 17.69	36.30 ^a^ ± 9.86
Dietary fibre (g/day)	15 g/1000 kcal (30–36 g/2000–2400 kcal)	30.38 ^a^ ± 2.74	32.34 ^bc^ ± 4.14	30.05 ^ac^ ± 3.47	33.00 ^bc^ ± 5.14	31.75 ^ab^ ± 6.97	28.80 ^a^ ± 1.93	29.46 ^a^ ± 2.60	26.75 ^a^ ± 2.88	32.88 ^bc^ ± 11.48	37.30 ^c^ ± 2.95
Salt (g/day)	5 g	6.58 ^d^ ± 0.68	3.91 ^a^ ± 0.74	7.94 ^e^ ± 1.92	5.00 ^abc^ ± 0.94	12.83 ^f^ ± 1.15	5.27 ^bc^ ± 0.13	5.96 ^cd^ ± 1.52	5.18 ^bc^ ± 0.38	4.26 ^a^ ± 0.73	4.37 ^ab^ ± 0.42

Different letters in the same row indicate statistically significant differences (*p* < 0.05).

**Table 2:** Compliance of energy and nutrient parameters of hospital standard diets with dietary recommendations. (green = meets recommendation; red = does not meet recommendation).

	Hospitals									
	H1	H2	H3	H4	H5	H6	H7	H8	H9	H10
Energy										
Protein (g)										
Protein (% of energy)										
Fat (g)										
Fat (% of energy)										
SFA (g)										
SFA (% of energy)										
Carbohydrates (g)										
Carbohydrates (% of energy)										
Sugars (mono- and disaccharides) (g)										
Fibre (g)										
Salt (g)										

Hospitals are coded as H1–H10, where each code represents one hospital participating in the assessment. Green fields indicate compliance with the recommendation, while red fields indicate that the parameter does not meet the recommended level.

**Table 3:** Assessment of the Compliance of Hospital Menus with the Requirements of the Standard Diet. (as part of the “Good Meal in the Hospital” programme) [18].

			Meets Requirement (Yes [🟢]/No [🔴])									
No.	Evaluation Criterion	Requirements	H1	H2	H3	H4	H5	H6	H7	H8	H9	H10
**1**	Number of meals per day	3–5 meals/day	🟢	🟢	🟢	🟢	🟢	🟢	🟢	🟢	🟢	🟢
**2**	Sensory and technological variety	Adequate diversity of flavours, colours, textures	🟢	🟢	🟢	🟢	🟢	🟢	🟢	🟢	🟢	🟢
**3**	Beverage with each meal	Each meal must include water/beverage; drinks ≤ 10 g sugars per 250 mL	🟢	🟢	🟢	🟢	🟢	🟢	🟢	🔴	🟢	🟢
**4**	Vegetables and fruits with each meal	≥400 g/day (excluding potatoes and sweet potatoes); predominance of vegetables; some raw	🔴	🟢	🟢	🟢	🟢	🔴	🟢	🔴	🟢	🔴
**5**	Whole-grain products	≥1 meal/day should include whole-grain cereals	🟢	🟢	🔴	🟢	🟢	🔴	🟢	🔴	🔴	🟢
**6**	Dairy or fortified plant-based alternatives	Present in ≥2 meals/day	🔴	🟢	🔴	🟢	🟢	🟢	🟢	🟢	🔴	🟢
**7**	Daily protein source	At least one serving of meat, eggs, legumes, or plant-based protein alternatives daily	🟢	🟢	🟢	🟢	🟢	🟢	🟢	🟢	🟢	🟢
**8**	Legumes	≥3 times per 10-day menu cycle	🔴	🔴	🔴	🔴	🔴	🔴	🟢	🔴	🟢	🟢
**9**	Fish	≥3 times per 10-day menu cycle (preferably marine fish)	🔴	🟢	🔴	🔴	🔴	🔴	🟢	🟢	🟢	🔴
**10**	Culinary techniques	Boiling, steaming, stewing, baking; frying ≤3 times per 10 days	🟢	🟢	🟢	🟢	🟢	🟢	🟢	🟢	🟢	🟢
	Score		6	9	6	8	8	6	10	6	8	8

Hospitals are coded as H1–H10, where each code represents one hospital participating in the assessment.

## Data Availability

The original contributions presented in the study are included in the article, further inquiries can be directed to the corresponding author.
